# Reconstruction after external hemipelvectomy using tibia-hindfoot rotationplasty with calcaneo-sacral fixation

**DOI:** 10.1186/1477-7800-5-1

**Published:** 2008-01-21

**Authors:** George YX Kong, Hannes A Rudiger, Eugene TH Ek, Wayne A Morrison, Peter FM Choong

**Affiliations:** 1 Department of Orthopaedic Surgery, St. Vincent's Hospital, Melbourne, Australia; 2 Department of Plastic Surgery, St. Vincent's Hospital, Melbourne, Australia

## Abstract

**Background:**

External hemipelvectomy is associated with high post operative morbidity and a poor functional outcome. We aim to explore a reconstruction technique to improve function and post operative appearance for patients who undergo external hemipelvectomy.

**Case presentation:**

We present a Case where extensive cancer involvement of pelvis and femur was managed with a novel surgical technique, which involved a calf sparing modified anterior flap hemipelvectomy combined with rotationplasty of the spared calf and fixation of calcaneus to the sacrum, thereby recreating a new thigh stump.

**Conclusion:**

Tibia-hindfoot rotationplasty result in good functional outcome and appearance for selected patients undergoing external hemipelvectomy with unaffected external iliac and femoral vessels.

## Background

Despite the advent of increasingly effective chemotherapy and limb-salvage surgery, external hemipelvectomy may remain the only surgical treatment for extensive tumors arising from the hip or pelvis. It is also a potential life-saving procedure for patients with massive pelvic trauma and uncontrollable sepsis of the lower limb. Unfortunately, external hemipelvectomy is often associated with high morbidity and poor quality of life for patients. Postoperative appearance following conventional external hemipelvectomy is also often poor, resulting in body image issues.

We here present a novel reconstructive technique aiming to improve function and appearance for patients requiring external hemipelvectomy. This technique is applicable in selected patient with unaffected external iliac and femoral vessels (i.e. most patients who would otherwise qualify for an anterior flap hemipelvectomy). The reconstruction involves a modified anterior flap hemipelvectomy with resection of the femur and hemipelvis but preservation of below knee structures. The calf with its vascular supply is rotated by 180 degrees. The fore- and mid-foot are then resected. The calcaneus is then fixed to the osteotomy site at the sacrum, thereby recreating a new thigh stump.

## Case presentation

**Figure 1:**
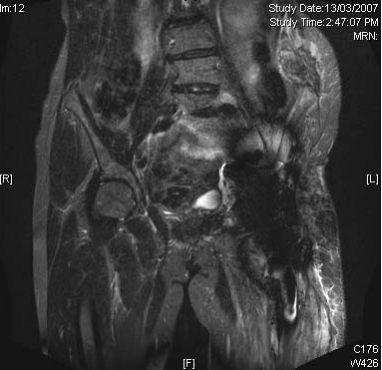
Pre-Operative MRI coronal view of Case Patient showing local cancer recurrence involving left pelvis and surrounding the saddle prosthesis.

68 year-old female presented to our unit with a 10 year history of recurrent malignant fibrohistiocytoma involving left pelvis and femur. She was initial treated 10 years ago with radical radiotherapy, following which she developed local recurrence after 5 years. Due to the extensive involvement of hemipelvis and proximal femur, a type II hemipelvectomy and reconstruction with saddle prosthesis was performed. The surgery was complicated by methicillin-resistant staphylococcus aureus (MRSA) infection for which she was managed with surgical washout and long term suppressive antibiotic treatment. She represented to our unit 5 years following saddle-prosthesis surgery with local tumor recurrence within the pelvis and surrounding the prosthesis. (Figure 1) No systemic recurrence was detected on chest CT, whole body Tc^99^-bone scan or Thallium scan. Following a course of neoadjuvant radiation therapy, surgical resection and reconstruction was performed as detailed below.

## Operative Technique

**Figure 2:**
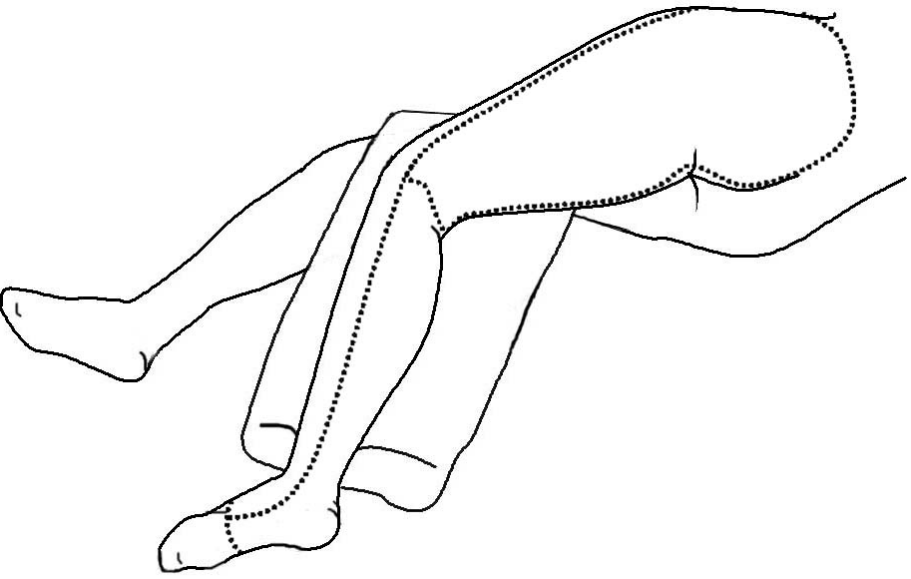
Diagrammatic outline of skin incision (posterior lateral view).

The resection technique was a variant of the anterior flap hemipelvectomy described by Sugarbaker and Chretien [1]. The patient was positioned in a floppy semi-lateral position on the operation table. Skin incision was made as shown. (Figure 2) In brief, skin incision commenced proximal to the anterior superior iliac spine aiming posterior and parallel to the iliac crest. The incision then curved around the posterior superior iliac spine and ran inferiorly approximately 5 cm lateral to the anus. It then ran along the medial aspect of the thigh towards the medial femoral condyle, traversed the popliteal fossa to the lateral condyle and returned to the anterior superior iliac spine along the lateral aspect of the thigh.

Dissection at the level of pelvis and proximal thigh was performed with the aim to preserve the calf. Briefly, deep dissection commenced proximal to the iliac crest, where the abdominal wall musculature was divided. The internal aspect of the iliac muscle and the sacroiliac joint were dissected, thereby dividing the sacral plexus.

A musculocutaneous anterior thigh flap comprising quadriceps and parts of the adductor compartment was then elevated based superficially. Along the medial thigh incision, the superficial femoral artery and vein were identified at the level of the adductor canal underneath the sartorius muscle and the vessels were skeletonized distally into the popliteal fossa keeping the blood supply to both heads of the gastrocnemius intact to for the basis of a filleted musculoskeletal calf flap. The tibial nerve and the common peroneal nerve were divided at this level. The knee joint was then disarticulated and the patella resected. The musculocutaneous thigh flap is further developed by elevating the quadriceps muscles and adductor muscles from the femur. The muscular flap, which is in continuity with the calf (superficial femoral and popliteal vessels and saphenous nerve intact), is then retracted anteriorly to allow adequate access to the hip and pelvis. The adductors are detached from the pubis and the symphysis is divided.

From posterior, the medial origin of the gluteus maximus was elevated from the sacrum. An osteotomy was performed through sacrum from the greater sciatic notch laterally to approximately 1 cm medial to the sacroiliac joint. Ileo-lumbar, sacro-tuberal and sacro-spinal ligaments are divided. Under external rotation of the hemipelvis, the hemipelvis, tumour mass and femur were delivered *en-bloc*.

### Preparation of rotational flap

The rotational tibia-hindfoot flap was supplied by the superficial femoral artery and vein. A skin incision was made from the lateral end of the popliteal crease parallel to the fibula down to the posterior aspect of the lateral malleolus. A fillet flap consisting of superficial posterior muscle compartment and overlying skin was developed from the lateral calf incision (vascularized by perforators through the soleus and gastrocnemius muscles). A fillet flap of the foot was made based on the dosalis pedis pedicle dorsally and the posterior tibial pedicle medially. The ankle and subtalar joints were preserved. The osseous forefoot was amputated through the transverse tarsal joint (Chopart).

**Figure 3:**
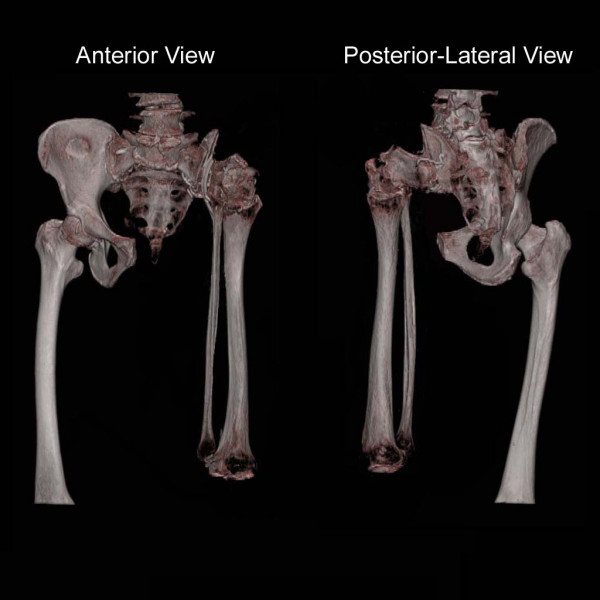
Post operative 3D reconstruction of pelvis and neo-thigh. Note the 180 degree rotation of tibia-fibula and fixation of calcaneus to ileosacral osteotomy site.

The tibia-hindfoot flap was then rotated such that the lateral aspect of the calcaneus came in contact with the ileosacral osteotomy. The calcaneus was the fixed to the sacrum with two sternal wire cerclages. (Figure 3) The quadriceps muscle flap was then sutured to the fillet flap on the calf, thereby forming a neo-thigh stump. Surgery was completed with skin closure.

## Post Operative Course

**Figure 4:**
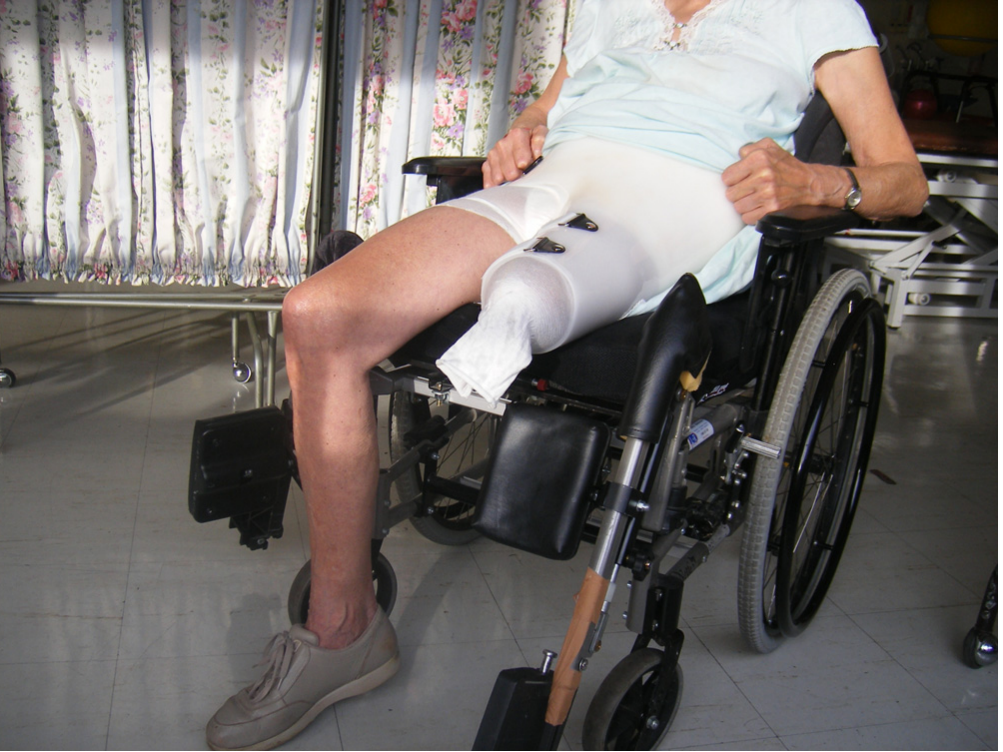
Clinical appearance of Case Patient 2 months following operation. Case Patient was able to sit upright comfortably with brace and was able to ambulate independently with crutches.

Postoperatively, the patient remained non-weight bear on the neo-thigh. The stump was held in a flexed position (70 degrees) in a brace to allow for a pseudo-arthrosis to develop between the calcaneus and the ileosacral osteotomy. Two weeks following the operation, the patient developed superficial wound breakdown at the posterior aspect of her buttock. After surgical debridement and wound closure, no further complications occurred. No phantom limb pain developed. By two months post op, patient was able to sit upright comfortably and ambulate with two crutches. (Figure 4)

## Discussion

Wide resection remains an important principle in the treatment of bone and soft tissue sarcomas. Despite the fact that the majority of patients with pelvic sarcomas can be safely treated with chemotherapy and limb-salvage surgery, [2] in a number of patients external hemipelvectomy is still required to achieve wide margins. Techniques used for soft tissue coverage following external hemipelvectomy include the posterior flap and the anterior flap closure [3,4]. These techniques aim to achieve tissue coverage but is often associated with high morbidity and poor functional outcomes [5,6]. Post operatively the majority of patients are either unable to ambulate without the use of crutches (80%) or remaining wheelchair bound or bed bound (15%); only around 5% of patients are eventually able to ambulate with prosthesis [7]. Well-fitted prosthesis for post-external hemipelvectomy patients is difficult to produce and the energy requirement to ambulate with prosthesis is very high. For patients who had external hemipelvectomy even the ability to sit upright can be difficult without a well-fitted sitting socket because of the lack of an ischium. Psychological issues surrounding body image following external hemipelvectomy is also significant and requires extensive pre and pos operative counseling [8].

Reconstructive surgery in our Case involved the combination of external hemipelvectomy with a rotation tibia-hindfoot flap resulting in the fixation of calcaneus to the ileosacral osteotomy. The calcaneus tubercle functions as a neo-ischium and the tibia-hindfoot flap functions as a neo-thigh. This allows the patient to sit upright without the aid of a sitting socket. Cosmetically, the surgery results in an apparent above knee stump.

*Peterson et al *had previously described two cases of tibia-hindfoot rotationplasty with calcaneopelvic arthrodesis for infective complications involving the proximal femur. In both cases no parts of the bony pelvis had been resected and the calcaneus could be fixed to the acetabulum. Movement was allowed as a result of retained tibiotalar and subtalar joints. Post operatively these patients were able to tolerate full weight bear with the use of a standard above knee amputation prosthesis [9]. Similar tibial turn-up procedure to manage distal femoral bone loss has also been described in pediatric patients with good functional result [10,11]. The surgical resection in our Case was much more extensive, with the removal of entire hemi-pelvis and femur. Despite this the patient in our Case has made excellent recovery with good sitting function and cosmetically satisfactory result.

## Conclusion

The Case illustrates that the use of the novel technique of tibia-hindfoot rotationplasty can result in good functional outcome and appearance for selected patients undergoing external hemipelvectomy with unaffected external iliac and femoral vessels.

## Competing interests

The author(s) declare that they have no competing interests.
